# In Silico Development of a Targeted Small Interfering RNA (siRNA) Sequence to Silence Goblet Cell Mucin-5AC Overexpression in Cystic Fibrosis

**DOI:** 10.7759/cureus.97562

**Published:** 2025-11-23

**Authors:** Jeevpreet Ahluwalia, Aarush Katakam, Nehal Revuri, Sanjith Satish

**Affiliations:** 1 Medicine, The Innovative STEMagazine, College Station, USA; 2 Neurological Surgery, The Innovative STEMagazine, College Station, USA

**Keywords:** computational modelling, molecular dynamic, novel therapeutic approaches, rmsd, sirna

## Abstract

Cystic fibrosis (CF) is characterized by persistent airway obstruction, frequent infection, and worsening lung function. Although CFTR channel-modulating therapy has improved outcomes, mucus hypersecretion morbidity remains present in most patients. Mucin 5AC (MUC5AC) is the main actor, a gel-forming mucin whose expression is highly upregulated in airway inflammation. Mucolytic and anti-inflammatory drug therapy reduces symptoms but does not prevent overproduction of mucin.

We present here the in silico design and computational evaluation of a chemically synthesized small interfering RNA (siRNA) against MUC5AC mRNA. The duplex candidate has four modifications, 5′ phosphorylation, site-specific 2′-O-methylation, amino-terminal conjugation, and hexadecyl lipid conjugation, aimed at promoting Argonaute 2 (Ago2) loading, increasing nuclease resistance, suppressing innate immune recognition, and enhancing membrane interaction. The computational approach was validated using a GL2 positive control and an inverted negative control sequence, which showed the expected stable and unstable binding behaviors, respectively. In 20-ns molecular dynamics simulations on OpenMM, the experimental siRNA-Ago2 complex showed stable binding (root-mean-square deviation (RMSD) 1.44±1.35 Å) comparable to the GL2 positive control (0.26±0.77 Å). The negative control complex, on the other hand, showed structural instability (11.94±2.29 Å). In addition, the experimental complex achieved energetically favorable binding with a converged binding energy of -11.2±0.21 kcal/mol at approximately 12.5 ns, demonstrating stable complex formation comparable to the positive control complex (-41.9±0.27 kcal/mol at about 12 ns). In contrast, the negative control failed to converge, oscillating around 0.2±0.9 kcal/mol without achieving definitively favorable binding.

In summation, this paper attempts to provide a computational basis for the development of MUC5AC-targeted siRNA as a therapeutic treatment for CF.

## Introduction

Cystic fibrosis (CF) is an autosomal recessive disease caused by mutations in the CFTR gene, which encodes a chloride and bicarbonate channel essential for airway hydration and epithelial homeostasis [1]. CFTR modulators, such as elexacaftor-tezacaftor-ivacaftor, have improved survival and lung function [2], but these therapies do not fully address downstream symptoms. Many patients continue to experience chronic infection, inflammation, and progressive lung destruction [3]. Additionally, approximately 10% of individuals carry mutations not responsive to current modulators, highlighting the need for alternative treatments [4].

Airway mucus plays a central role in CF pathophysiology. Altered ion transport results in airway surface liquid dehydration, impaired mucus clearance, and formation of mucus plaques [5]. The relative amounts and biochemical properties of secreted mucins are critical for disease progression. MUC5B, the dominant gel-forming mucin in normal airways, facilitates efficient mucus clearance. By contrast, Mucin 5AC (MUC5AC) expression is normally low but is strongly induced by proinflammatory mediators such as IL-1β and IL-17A via the NF-κB signaling pathway [6]. Clinical evidence indicates that MUC5AC increases nearly 10-fold in sputum during CF exacerbations and correlates with airflow limitation and neutrophil elastase activity [7]. Excessive MUC5AC contributes to pathological mucus viscoelasticity, impairs clearance, and amplifies infection and inflammation [8].

Current therapies do not adequately address mucin hypersecretion. Recombinant human DNase (dornase alfa) reduces DNA-mediated viscosity but does not affect mucin gene expression [9]. Mucolytics and hypertonic saline modestly modify mucus properties but require continuous administration and have limited efficacy. Anti-inflammatory therapies, such as high-dose ibuprofen, reduce neutrophilic damage but do not suppress mucin upregulation [10]. Direct inhibition of MUC5AC expression represents an untested therapeutic approach with potential to interrupt the cycle of mucus accumulation and inflammation.

A search of ClinicalTrials.gov revealed no ongoing or completed trials targeting MUC5AC expression in CF, and no siRNA therapies currently in clinical trials appear to address downstream hypersecretion beyond ion transport (search conducted October 2025).

RNA interference (RNAi) is a natural post-transcriptional gene regulation process in which small interfering RNAs (siRNAs) guide Argonaute-containing RNA-induced silencing complexes (RISC) to complementary mRNA, triggering cleavage and degradation [11]. Synthetic siRNAs have been developed into a new class of therapeutics, including drugs approved for transthyretin amyloidosis, hypercholesterolemia, and acute hepatic porphyria [12,13]. Local pulmonary delivery is particularly advantageous as it enables direct targeting of the airway epithelium with reduced systemic exposure [14].

Despite this promise, pulmonary siRNA therapy faces challenges. Thick CF mucus inhibits penetration of naked siRNA, while extracellular nucleases and activation of Toll-like receptors compromise stability and safety [15,16]. Chemical modifications have been incorporated to improve pharmacologic properties, including 2′-O-methylation for immunosuppression, terminal phosphorylation to facilitate Argonaute 2 (Ago2) loading, and lipid conjugation to enhance cell entry [17,18]. These advances support the rational design of siRNAs optimized for the CF airway environment.

We hypothesize that a siRNA can be designed with high specificity and stability to target MUC5AC transcripts and prevent thick mucus formation.

In this study, we used computational methods to design and characterize an optimized, chemically modified MUC5AC siRNA. Both thermodynamic criteria and off-target elimination guided sequence selection, followed by four synergistic chemical modifications to enhance stability and efficacy. Complex stability and conformational adaptability under near-physiological conditions were assessed via docking simulations with Ago2 and explicit-solvent molecular dynamics.

These computational findings aim to lay the groundwork for MUC5AC knockdown as a therapeutic approach for mucosal hypersecretion in CF, complementing CFTR modulators.

## Materials and methods

Computational design of short interfering RNAs for the human MUC5AC transcript (GenBank: NM_001304359.1) was performed under limiting conditions to selectively favor biologically active duplexes using the siDirect 2.0 algorithm [19]. Six hundred candidate siRNA strands were identified, of which 30 remained after filtering for melting point, reduced off-target silencing, and predicted silencing efficacy. Melting points were constrained to 21.5°C to enable unwinding at physiological temperatures. Basic Local Alignment Search Tool (BLAST) searches against the human transcriptome were performed for each candidate, and sequences with more than 80% identity over 15 consecutive nucleotides were removed to minimize off-target silencing [19]. The final filtering was conducted using siPred, selecting the sequence with the highest predicted silencing efficacy score (0.1-1, higher indicating greater efficacy) after individually inputting the remaining 30 siRNA sequences into siPred. The final duplex had an siPred score of 0.971, indicating high potency for MUC5AC silencing. It was 21 nucleotides in length, with two-nucleotide 3′ overhangs on both strands, 47.6% guanine-cytosine (GC) content, and thermodynamic asymmetry favoring guide strand incorporation into the RISC.

Guide strand

5'-pUUAUGCAACAGAUmUmGmGmCmCGUG-3'

Passenger strand

5'-pNH2-mCmGmGmCmCAAUCUGUUGCAUAAAU-C16-3

The final siRNA duplex was chemically modified with four synergistic alterations to optimize pharmacological stability and intracellular activity. The guide strand was phosphorylated at the 5′ terminus to facilitate high-affinity docking into the Argonaute 2 (Ago2) middle (MID) domain. Selected ribose positions were substituted with 2′-O-methyl groups to confer nuclease resistance and avoid activation of innate immune receptors, including Toll-like receptors 7 and 8. An amino-terminal modification reinforced hydrogen bonding networks in the Ago2 binding cleft, and the 3′ end was conjugated with a C16 hexadecyl lipid to enhance membrane association and retention [18,20].

To simulate protein-RNA interactions, the human Ago2 crystal structure (PDB ID: 4W5N, 2.3 Å) was obtained and prepared for simulations. Missing atoms were reconstructed, protonation states adjusted to physiological pH, and the structure minimized under the CHARMM36 (Chemistry at HARvard Macromolecular Mechanics 36) force field. The target siRNA was constructed using AlphaFold-derived RNA pipelines and minimized in AMBER (Assisted Model Building With Energy Refinement) with the ff14SB protein force field and OL3 RNA parameters. The 2′-O-methyl ribose residues and lipid conjugates were parameterized in Antechamber and integrated into OL3 topologies [21,22].

Docking of the siRNA to Ago2 was performed using HADDOCK 2.4. This involved rigid-body sampling of 1000 poses, semi-flexible refinement of the top 200 complexes, and explicit-solvent refinement in TIP3P water. Active residues included those forming the 5′ phosphate binding pocket (Lys309, Arg315, Tyr529, and Phe647) and those orienting the siRNA seed region (Gln757, Arg761, and Lys786). The top 10 scoring docked complexes were selected for molecular dynamics simulations [23,24].

All-atom, explicit-solvent molecular dynamics simulations were performed using OpenMM version 7.7 on NVIDIA H200 GPUs. Three systems were modeled: the experimental MUC5AC siRNA-Ago2 complex, a positive control GL2 siRNA-Ago2 complex, and an inverted negative control GL2 duplex. All systems were solvated in cubic TIP3P water boxes with 10 Å padding, neutralized with counterions, and supplemented with 150 mM NaCl to mimic intracellular ionic conditions. Following energy minimization, the systems were heated from 0 to 310 K at constant volume and equilibrated at 1 atm using a Parrinello-Rahman barostat. Each system underwent a 20 ns production run with a 2 femtosecond timestep, temperature controlled by a Nosé-Hoover thermostat, long-range electrostatics by particle mesh Ewald, and hydrogen-involving bonds constrained by SHAKE [3,25,26]. Production runs were limited to 20 ns due to computational resources and MD inexperience. Shorter simulations may not guarantee convergence in large RNA-protein complexes; a convergence graph was generated to validate stability.

Trajectory analysis was conducted using MDAnalysis and CPPTRAJ. Structural stability was assessed via root-mean-square deviation (RMSD) relative to the initial docked conformation. Atomic flexibility was calculated to quantify residue-level mobility. Statistical convergence was evaluated through block averaging of RMSD profiles, and the experimental complex was compared with controls to assess relative stability and flexibility [4,5,27].

Graphs were generated using Matplotlib [28].

**Figure 1:**
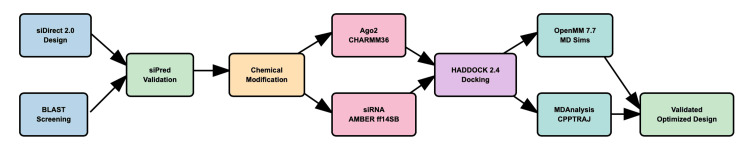
Computational pipeline of siRNA design and testing siRNA, small interfering RNA; BLAST, Basic Local Alignment Search Tool; CHARMM36, Chemistry at HARvard Macromolecular Mechanics 36; AMBER, Assisted Model Building With Energy Refinement

Figure 1 summarizes the computational pipeline.

## Results

**Figure 2:**
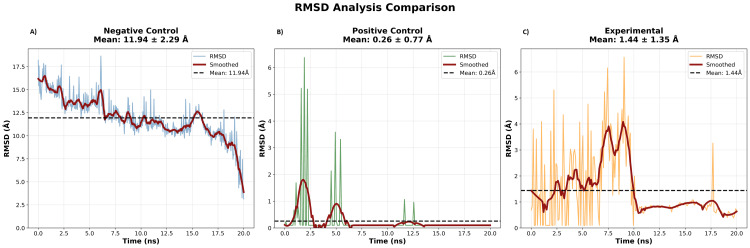
RMSD of Ago2-siRNA complexes over time (A) Negative control, (B) positive control, and (C) experimental. RMSD plots show structural changes over 20 ns of simulation. Stable complexes are indicated by lower RMSD values and flat trajectories, while large fluctuations or drifts reflect conformational instability. RMSD, root-mean-square deviation; siRNA, small interfering RNA; Ago2, Argonaute 2

Figure 2 shows the behavior of the three siRNA-Ago2 complexes during 20 ns of molecular dynamics simulation. RMSD measures how far the structure deviates from its initial position. Flat and lower curves indicate a stable complex, while high or erratic curves indicate instability. The negative control never stabilized; its RMSD values fluctuated widely from 3 Å to 19 Å, with a mean of 11.94±2.29 Å, reflecting ongoing duplex rearrangement. The positive control stabilized rapidly after minor adjustments at the beginning of the simulation, with a mean of 0.26±0.77 Å throughout. The experimental siRNA behaved similarly to the positive control, fluctuating slightly at first and then stabilizing, with a mean RMSD of 1.44±1.35 Å and a few deviations greater than 6 Å.

**Figure 3:**
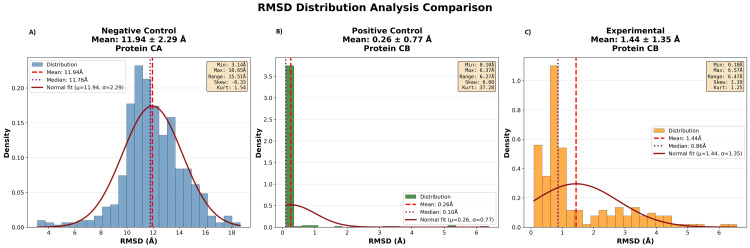
RMSD distribution profiles of the simulated Ago2-siRNA complexes (A) Negative control, (B) positive control, and (C) experimental. RMSD distributions indicate the frequency of specific structural deviations during the simulation. Narrow peaks represent stable conformations, while broader distributions reflect greater conformational variability. RMSD, root-mean-square deviation; siRNA, small interfering RNA; Ago2, Argonaute 2

Figure 3 shows the RMSD distributions of the three complexes. RMSD distributions indicate the frequency and extent of structural deviations in the binding conformation during the simulation. The negative control had a peak at 12 Å, indicating the complex sampled many unstable conformations rather than adopting a stable structure. The positive control displayed a narrow peak at 0.5 Å, reflecting a stable single conformation. The experimental siRNA had a narrow peak around 1.5 Å, with most conformations within 2 Å and rare deviations beyond 4 Å.

**Figure 4:**
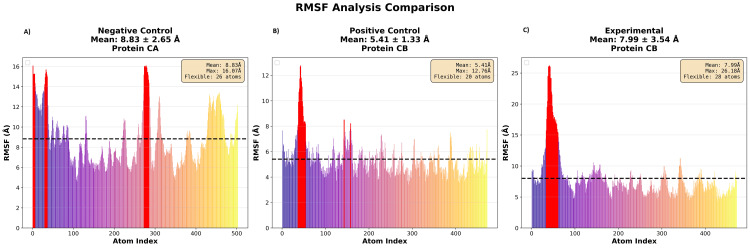
RMSF of Ago2-siRNA complexes F(A) negative control, (B) positive control, and (C) experimental. RMSF (reported in Ångströms) quantifies the average motion of each atom or residue over the trajectory. Higher peaks indicate flexible or mobile regions, while lower values reflect more rigid, stable regions. RMSF, residue-level atomic flexibility; siRNA, small interfering RNA; Ago2, Argonaute 2

Figure 4 shows the root-mean-square fluctuation (RMSF) of the three complexes, which measures residue flexibility during the simulation. The negative control was highly flexible, with a mean RMSF near 9 Å and a maximum above 16 Å, particularly at terminal and loop regions. The positive control was the least flexible across almost all residues. The experimental siRNA was intermediate, with an average RMSF of approximately 8 Å and a localized peak exceeding 26 Å. Notably, these fluctuations were confined to some seed nucleotides of the siRNA and did not propagate across the entire structure. In contrast, the negative control showed flexibility across many residues, indicating greater destabilization.

**Figure 5:**
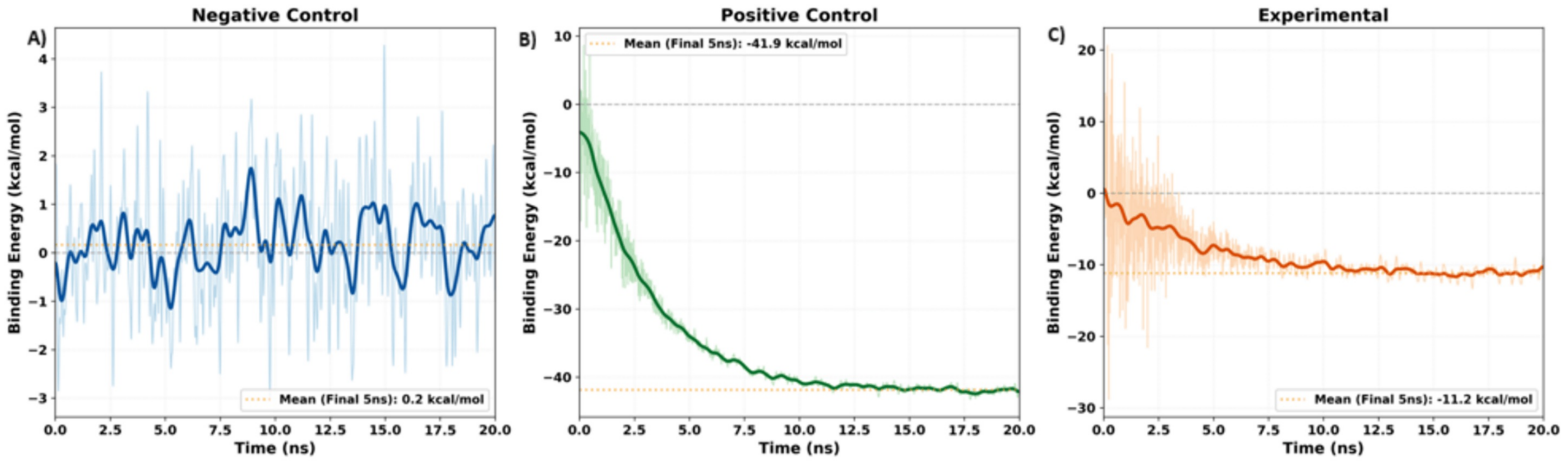
Binding energy convergence profiles of Ago2-siRNA complexes during 20 ns of molecular dynamics simulation (A) negative control, (B) positive control, and (C) experimental. Negative binding energies indicate energetically favorable complex formation, with more negative values corresponding to stronger binding affinity. siRNA, small interfering RNA; Ago2, Argonaute 2

Figure 5 shows the convergence graphs of the three complexes, presenting binding energy in kcal/mol over simulation time in nanoseconds. The positive control converged to approximately -41.9±0.27 kcal/mol at 12 ns and maintained stable binding energy throughout the simulation. The experimental siRNA exhibited similar convergence, stabilizing at 12.5 ns at -11.2±0.21 kcal/mol. In contrast, the negative control did not converge, oscillating around 0 kcal/mol with a mean of 0.2±0.9 kcal/mol and failing to achieve energetically favorable binding, remaining unstable even at the 20 ns mark.

Table 1 summarizes the experimental values. 

**Table 1:** Molecular dynamics simulation parameters for siRNA-Ago2 complexes RMSD, RMSF, and binding energy values from 20 ns simulations of the negative control (Protein CA), positive control (Protein CB), and experimental MUC5AC-targeting siRNA (Protein CB). Values are reported as mean ± SD. RMSD, root-mean-square deviation; RMSF, residue-level atomic flexibility; siRNA, small interfering RNA; Ago2, Argonaute 2; MUC5AC, Mucin 5AC

Parameter	Negative Control (Protein CA)	Positive Control (Protein CB)	Experimental (Protein CB)
RMSD Trajectory (Å)			
Mean±SD	11.94±2.29	0.26±0.77	1.44±1.35
Smoothed Mean	11.94 Å	0.26 Å	1.44 Å
Observed Range	~3-19 Å	~0-3.5 Å	~0-6.5 Å
Stabilization Behavior	Never stabilized, continuous fluctuation	Stabilized quickly after initial adjustment	Initial fluctuation, stabilized after equilibration
RMSD Distribution			
Mean	11.94 Å	0.26 Å	1.44 Å
Median	10.51 Å	0.27 Å	6.47 Å
Range	15.15 Å	6.22 Å	6.74 Å
Kurtosis	1.54	37.28	1.35
Distribution Peak	~12 Å	~0.5 Å	~1 Å
Peak Width	Broad (4-18 Å)	Extremely narrow (~0-0.7 Å)	Narrow (0-2 Å)
Normal Fit	μ=11.94, σ=2.29	μ=0.26, σ=0.77	μ=1.44, σ=1.35
RMSF (Å)			
Mean±SD	8.83±2.65	5.41±1.32	7.95±3.83
Maximum	16.07 Å	12.76 Å	26.19 Å
Flexible Atoms/Regions	26 atoms	28 atoms	28 atoms
Flexibility Pattern	High flexibility across entire structure	Lowest overall flexibility	Localized high peaks (atom ~25), moderate elsewhere
Notable Features	Multiple peaks >12 Å distributed throughout	Most residues <8 Å	Extreme spike at a specific seed region residue
Binding Energy (kcal/mol)			
Mean±SD	0.2±0.9	-41.9±0.27	-11.2±0.21
Convergence Time	No convergence	~12 ns	~12.5 ns
Production Phase	N/A - never converged	12-20 ns	12.5-20 ns
Converged Value	Oscillates around 0	-41.9 kcal/mol	-11.2 kcal/mol
Convergence Assessment	No convergence	Converged	Converged

## Discussion

The results indicate that the MUC5AC-targeting siRNA forms a structurally stable complex with Ago2.

RMSD plots demonstrate the structural behavior of the complexes. The negative control never stabilized, maintaining high RMSD values throughout the simulation. The experimental siRNA stabilized after initial equilibration, achieving a structural deviation range similar to the GL2 positive control. RMSD distribution analysis supports this conclusion, showing that the experimental and positive control complexes occupied low-RMSD values, whereas the negative control remained in a broad high-RMSD range.

The binding energy plot further supports the stability of the experimental complex. The mean converged binding energy of the experimental complex (-11.2±0.21 kcal/mol at approximately 12.5 ns) was less negative than the positive control (-41.9±0.27 kcal/mol at approximately 12 ns), but both demonstrated energetically favorable binding. In contrast, the negative control never achieved energetically favorable binding, oscillating around 0.2±0.9 kcal/mol in the last 5 ns of the simulation (albeit the negative control never quite converged, see limitations). The small standard deviations in the experimental and positive control complexes (±0.21 and ±0.27 kcal/mol) indicate convergence, whereas the high standard deviation of the negative control (±0.9 kcal/mol) reflects instability.

These results confirm and extend the findings of Ramachandran et al., demonstrating that chemical modifications enhance siRNA stability and delivery efficiency in polarized airway epithelia by reducing degradation and enabling transfection across cellular barriers [15]. In agreement with previous molecular and biochemical evidence [12,15,28], low RMSD values and convergent conformations indicate that a stable siRNA-Ago2 interaction is essential for sustained RNA interference. The correspondence between structural stabilization and in vivo gene silencing efficacy [13,14] supports the expectation that chemical modifications enhance molecular stability.

These findings have several limitations. The study tested four modifications simultaneously without an unmodified control, making it impossible to determine which chemical alterations contributed to stability. Free energy calculations were not performed, so it is unclear whether the modifications confer more energetically favorable binding in addition to structural stability. The RMSF of the experimental complex was higher than that of the positive control, limiting conclusions about the effectiveness of siRNA binding to Ago2. All results are from computational simulations, so experimental validation is required to confirm Ago2 loading and MUC5AC silencing activity. Other potential sources of error include uncertainties in force field parameterization for modified residues and short trajectory lengths (20 ns), likely explaining why the negative control did not reach a stable conformation [17,21]. The absence of a convergent negative control reduces the robustness of comparisons with the experimental and positive control complexes.

Regarding limitations, the experimental complex showed higher RMSF (7.95±3.83 Å) than the positive control (5.41±1.32 Å) and was similar to the negative control (8.83±2.65 Å). Lower flexibility generally correlates with more stable Ago2 binding and consistent positioning of critical residues during assembly. The elevated RMSF in the experimental complex, confined to specific seed nucleotides rather than distributed across the structure like the negative control, may reduce binding strength, affecting recognition fidelity and RISC loading efficiency. Error bars were not included for RMSD and RMSF graphs, as each metric represents a single 20 ns trajectory; multiple simulations would be required to assess inter-system variability.

These gaps should be addressed in future research. Future studies should include an unmodified siRNA control group (optimized to the seed region) to isolate and quantify the contributions of chemical modifications. Longer molecular dynamics simulations (100 ns or more) should be conducted to allow all groups to converge and confirm that observed differences in Ago2 interactions are due to structural effects. Computationally, systematic analyses should examine each modification individually, while MM/PBSA (Molecular Mechanics/Poisson-Boltzmann Surface Area) or alchemical free energy calculations should determine Gibbs free energy changes between modified and unmodified complexes. Off-target prediction tools, such as Cas-OFFinder, should be used to identify potential unintended targets across the human transcriptome. Experimentally, Ago2 loading and mRNA cleavage assays are required to confirm functional activity. In cell-based CF models, siRNA efficacy should be tested against induced MUC5AC expression. For pulmonary delivery, hydrophobic lipid nanoparticles (HLNPs) effectively encapsulate modified duplexes, protect them from nuclease degradation, and facilitate intracellular uptake [7,29]. Delivery can be further optimized using mesh nebulizers that produce aerosols suitable for distal airway deposition, a requirement for clinical application in CF treatment [8,30].

## Conclusions

This study shows that a rationally designed siRNA targeting MUC5AC can form a stable, productive complex with Ago 2, providing initial computational evidence that mucin overexpression in CF can be silenced at the transcript level. By reducing MUC5AC expression, this gene therapy has the potential to decrease mucus viscosity, improve mucus clearance, and interrupt the cycle of airway obstruction, infection, and inflammation responsible for progressive lung damage. These findings demonstrate the potential of RNA interference delivered via the airway as an adjunct to CFTR modulators, enabling combined treatment strategies. Translation to in vitro testing should include biochemical confirmation of gene silencing, cellular assays of mucin regulation, and delivery systems capable of penetrating viscoelastic mucus. The computational model described here provides a clear framework for the rational design of mucin-targeted RNA therapeutics. Future administration via inhalation, delivering the siRNA in nebulized hybrid lipid nanoparticles (HLNPs), could provide an effective treatment usable outside of a clinical setting. Furthermore, this approach could be combined with CFTR modulators to enhance overall efficacy. Hypothetically, the successful application of this strategy could prevent hypersecretion and reduce hospitalization rates.
